# Development and validation of a simple risk score to predict gastroesophageal reflux disease after sleeve gastrectomy: a multicenter retrospective cohort study

**DOI:** 10.1097/JS9.0000000000003245

**Published:** 2025-08-20

**Authors:** Xiang Gao, Jiahao Li, Zhi Song, Weizheng Li, Lei Zhao, Youwu Wen, Henggui Luo, Tongli Yuan, Zhen Li, Pengzhou Li, Liyong Zhu

**Affiliations:** a Department of Gastrointestinal Hernia Surgery & Bariatric Metabolic Surgery, The Third Xiangya Hospital, Central South University, Changsha, Hunan, China; b Department of General Surgery, The First Affiliated Hospital of University of South China, Hengyang, Hunan, China; c Department of Gastrointestinal Surgery, Yueyang Central Hospital, Yueyang, Hunan, China; d Department of General Surgery, Xiangtan Central Hospital, Xiangtang, Hunan, China; e Department of General Surgery, Hunan Provincial Hospital of Traditional Chinese Medicine, Zhuzhou, Hunan, China; f Department of Hepatobiliary & Pancreatic Surgery, Zhongnan Hospital of Wuhan University, Wuhan, Hubei, China

**Keywords:** esophagogastric junction, gastroesophageal reflux disease, grading system, nomogram, sleeve gastrectomy

## Abstract

**Background:**

Laparoscopic sleeve gastrectomy (LSG) is the most commonly performed bariatric procedure worldwide. However, the development of gastroesophageal reflux disease (GERD) following LSG remains a significant clinical concern that can compromise long-term patient outcomes and satisfaction.

**Materials and methods:**

In this multicenter retrospective cohort study, a total of 968 patients were screened, of whom 851 met eligibility criteria and were included in the final analysis. Baseline characteristics, perioperative variables, and 12-month follow-up outcomes were collected. A predictive risk score for postoperative GERD was developed using LASSO regression and logistic modeling in a training cohort (n = 595) and subsequently validated in an independent cohort (n = 256). A nomogram was constructed based on the final model.

**Results:**

The study included 313 male and 538 female patients, with a mean age of 31.8 years and mean BMI of 38.4 kg/m^2^. Among the entire cohort, 27.3% developed GERD after LSG. Key associated factors included BMI, The American Foregut Society (AFS) classification stratifies EGJ integrity (AFS grade), preoperative reflux symptoms, distance from the angle of His, complete mobilization of the angle of His, and duration of postoperative PPI therapy. The final model incorporated 6 predictors and demonstrated high discrimination with an AUC of 0.948 in the training set and 0.912 in the validation set.

**Conclusion:**

We established and validated a robust, user-friendly risk score and nomogram to predict GERD after LSG. This tool enables early identification of high-risk patients and supports individualized perioperative decision-making, thereby enhancing long-term outcomes and optimizing patient care.

## Introduction

Laparoscopic sleeve gastrectomy (LSG) has become the leading bariatric procedure worldwide, accounting for roughly 62.5% of all primary bariatric operations recorded in the eighth International Federation for the Surgery of Obesity and Metabolic Disorders (IFSO) Global Registry Report 2023^[1]^, favored for its technical feasibility, short operative time, and effective weight loss and metabolic outcomes^[2-4]^. However, with its widespread use, there is increasing recognition of gastroesophageal reflux disease (GERD) as a common and clinically significant postoperative complication^[5,6]^. Unlike other adverse events, GERD not only affects patient satisfaction and quality of life but may also necessitate long-term medication use or even revisional surgery^[7]^. Its high prevalence and potential to undermine surgical benefits have made post-LSG GERD one of the most pressing unresolved challenges in current bariatric practice^[8]^.

GERD after LSG remains paradoxical, with some patients achieving symptom resolution and others developing de novo or aggravated reflux^[9–11]^. Reported incidence rates vary widely, with some studies indicating a nearly 32% in GERD risk after LSG 10 years follow-up^[12]^, while others suggest symptom improvement due to reduced gastric acid production and accelerated emptying^[13]^. This ongoing controversy highlights the lack of consensus regarding the impact of LSG on GERD, as well as the underlying mechanisms responsible. Despite extensive research, there remains no reliable, standardized tool to predict which patients are at risk for postoperative GERD. Most existing studies are limited by single-center design, small sample sizes, or lack of objective evaluation of esophagogastric junction (EGJ) function^[14,15]^. In particular, few studies have integrated endoscopic assessment, anatomical features, and perioperative variables into a unified risk prediction model.

Most published tools for forecasting GERD after sleeve gastrectomy draw on single-center, symptom-defined datasets and seldom undergo external validation, restricting their translational value^[15,16]^. Using the largest multicenter series reported to date and an endpoint verified by objective reflux testing, we derived and temporally validated a risk score that maintained accuracy across independent centers. Hence, we conducted a large multicenter retrospective cohort study involving 968 patients undergoing LSG. By incorporating clinical, anatomical, and procedural factors, we aimed to develop and validate a practical, evidence-based risk score for predicting new-onset GERD after LSG. This tool may support personalized surgical planning, improve preoperative counseling, and ultimately enhance postoperative outcomes. This cohort study has been reported in line with the STROCSS 2025 guidelines^[17]^

## Methods

### Patient information

**Figure 1.**
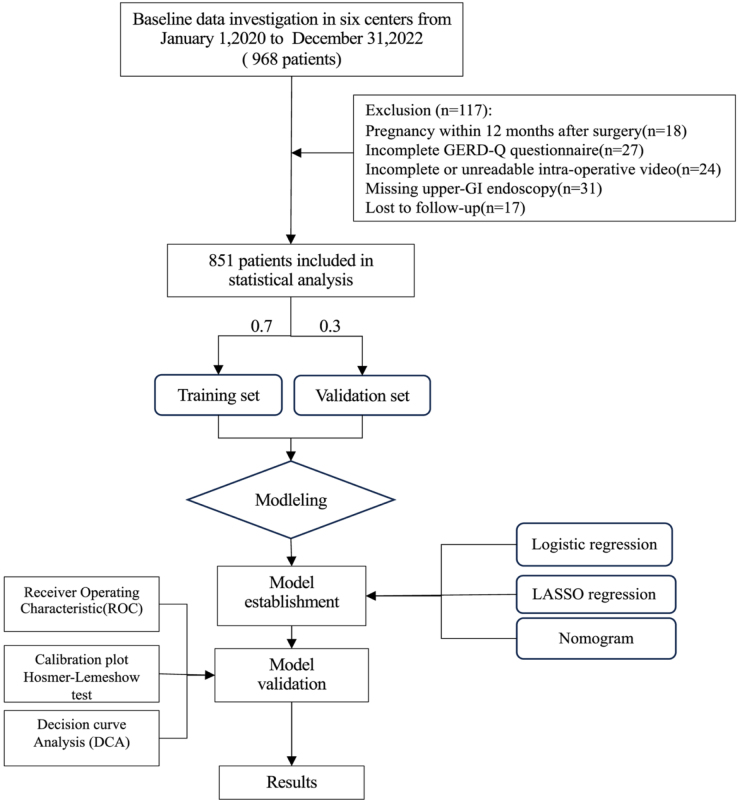
Flowchart of study design.

Between 1 January 2020 and 31 December 2022, 968 patients underwent baseline assessment; after applying the predefined exclusion criteria (Fig. 1), 851 were retained for statistical analysis. This study was initiated by Bariatric Metabolic Surgery Center at A and involved collaboration across six medical centers. The institutions that contributed ≥100 cases included the A, B, C, and D. The institutions with fewer than 100 cases included E and F. The study was approved by the Ethics Committee of A. The registration was completed in the Research Registry, and the unique identifying number (UIN) is researchregistry11054.

Patients were included if they met the surgical indications for LSG as defined in the 2019 Guidelines for Surgical Treatment of Obesity and Type 2 Diabetes in China. Additional inclusion criteria were a minimum of 12 months of postoperative follow-up and completion of a preoperative upper endoscopy. Exclusion criteria were: presence of mental or intellectual disabilities that could hinder postoperative follow-up; history of achalasia or prior gastroesophageal surgery; history of alcohol or drug dependence; and any preoperative diagnosis of GERD, including patients with endoscopically confirmed reflux or those requiring proton pump inhibitors for symptoms such as heartburn, pyrosis, or regurgitation.

### Data collection and follow-up

Follow-up assessments were conducted at baseline (hospital admission), and at 1 month, 3 months, and 12 months after surgery. Data were obtained through a combination of inpatient records, outpatient visits, and structured telephone interviews. All records were collected using a standardized structured template. Collected variables included demographic data (age, gender), lifestyle factors (smoking and alcohol use), and prior surgical history. Preoperative anthropometric measurements included weight, height, body mass index (BMI), waist circumference, and hip circumference. Medication history was reviewed, with particular attention to the use of non-steroidal anti-inflammatory drugs (NSAIDs) and proton pump inhibitors (PPIs).

**HIGHLIGHTS**

- A simple, clinically applicable nomogram was developed to predict GERD after sleeve gastrectomy.
- The model was based on a multicenter cohort of 851 patients and externally validated.
- Key predictors included BMI, AFS grade, reflux symptoms, distance from the angle of His, complete mobilization of the angle of His, and duration of PPI.
- The risk score enables personalized assessment and may help guide surgical decision-making.

Comorbidities were recorded, including diabetes (with duration), hypertension, chronic cough, benign prostatic hyperplasia, chronic constipation, Helicobacter pylori infection, anemia, and peptic ulcer disease. The presence of esophagitis and reflux symptoms was also documented. Intraoperative variables included the distance from the staple line to the pylorus and to the angle of His, bougie size (French units). Postoperative variables included PPIs use and percentage of total weight loss at 3 months.

### Gastrointestinal endoscopy

Preoperative upper gastrointestinal endoscopy was performed after an 8-hour fast and a 2-week discontinuation of PPIs or H2-receptor antagonists. Esophagitis was assessed using the Los Angeles (LA) classification. Postoperative endoscopy was conducted without PPIs or H2RA withdrawal. Each anonymized image data was reviewed independently and in a blinded fashion by two board-certified gastro-endoscopists (>10 years’ experience). In the event of discordant grading, a third senior endoscopist adjudicated the final score. This three-step procedure ensured consistent, high-quality EGJ evaluation across all centers and eliminated inter-observer bias.

The function of the esophagogastric junction was evaluated preoperatively using the American Foregut Society (AFS) classification system^[18]^, which is based on three parameters: axial length of the hiatus (L), maximal hiatus diameter (D), and the presence or absence of the gastroesophageal flap valve (F). EGJ integrity was graded as follows: Grade I: normal EGJ (L0, D1, F+); Grade II: partial disruption (L0, D1–2, F–); Grade III: moderate disruption (L0–2, D2–3, F–).

### Definition of GERD

GERD was evaluated based on typical reflux symptoms, the gastroesophageal reflux disease questionnaire (GERDQ), upper gastrointestinal (GI) endoscopy, and pH monitoring. Typical reflux symptoms of GERD include heartburn and regurgitation, and symptom severity was assessed using both an empirical reflux symptoms questionnaire and the validated GERDQ^[19]^. A GERDQ score ≥8 was considered indicative of a high probability of GERD.

Confirmed GERD was defined as any one of the following: (i) Los Angeles grade ≥B esophagitis on upper-GI endoscopy; (ii) pathological 24-h pH-impedance monitoring (acid-exposure time >6%); or (iii) continuous proton-pump-inhibitor therapy for ≥8 weeks prescribed for typical reflux symptoms^[20]^. Preoperative reflux-like symptoms without confirmed GERD were defined as follows: all remaining candidates completed the 6-item GERD-Q questionnaire at baseline. A GERD-Q score <8 (indicating no objective GERD) but with at least one weekly episode of heartburn or regurgitation was classified as preoperative reflux-like symptoms^[21,22]^. Any GERD-Q score ≥8 triggered an upper-GI endoscopy. If findings were unclear, two experienced gastroenterologists independently reviewed the symptoms and endoscopic images; disagreements were resolved by a third reviewer. Patients with isolated dyspeptic symptoms but no reflux features were not classified as GERD.

### Surgical technique

All procedures were performed by experienced bariatric surgeons using a standardized laparoscopic sleeve gastrectomy technique. Patients were placed in the supine reverse Trendelenburg position with legs apart. Pneumoperitoneum was established via a four-port approach. The greater curvature of the stomach was mobilized by dividing the gastrocolic, gastrosplenic, and gastrophrenic ligaments, as well as the short gastric vessels. The posterior fundus was fully freed to expose the angle of His. A bougie was inserted to calibrate the sleeve. Gastric transection began 2–6 cm from the pylorus and proceeded along the bougie to the angle of His, ensuring complete fundus resection. Staple lines were reinforced with sutures, and trocar sites were closed in layers.

Complete mobilization of the angle of His was defined as complete exposure of the left diaphragmatic crus and the angle of His by fully dissecting the fat pad surrounding the gastroesophageal junction. Operative procedures were documented using high-definition intraoperative video recordings. For each patient, two bariatric surgeons with over 10 years of specialized practice independently and blindly reviewed these recordings to evaluate the completeness of mobilization at the angle of His. In cases where the reviewers disagreed, a third senior bariatric surgeon independently reviewed the video and adjudicated the final assessment, ensuring consensus was reached for all cases.

### Statistical analysis

The analytic cohort contained no missing values for the variables used in model development or validation, so imputation was unnecessary. All statistical analyses were conducted using R software (version 4.2.3). The final cohort was randomly divided into a training set (70%) and an internal validation set (30%) using simple random sampling without replacement. To develop and validate a clinical prediction model for new-onset GERD following LSG, we used logistic regression analysis. The initial model development was performed using a training cohort. To identify significant risk factors for postoperative GERD, we initially included clinical variables in a LASSO (least absolute shrinkage and selection operator) logistic regression model. This method effectively addressed multicollinearity by applying L1 regularization, shrinking the coefficients of less informative predictors to zero and retaining only the most relevant features. Variable selection was guided by multivariable logistic regression analyses. Variables for the final model were selected using backward elimination, with a significance threshold set at *P* <0.05 to allow for the potentially important predictors. Multicollinearity among predictors was assessed using tolerance and variance inflation factor (VIF) values.

Model calibration was assessed using calibration plots, along with estimates of the calibration slope and intercept. Discrimination was evaluated by calculating the area under the receiver operating characteristic curve for both the development and validation cohorts. Internal validation of the derivation model was conducted using both 1000 bootstrap resamples and k-fold cross-validation to assess model stability and generalizability. For each bootstrap resample, the model was refitted and predictions for the original (out-of-bag) data were used to estimate optimism. Cross-validation was additionally applied to evaluate performance consistency across different data partitions. Discrimination and calibration metrics were adjusted for optimism. Model performance was summarized using the optimism-corrected AUC, calibration intercept, calibration slope.

Regression coefficients (β) from the final multivariable model were divided by the smallest |β| and rounded to the nearest integer to yield item scores; their sum constituted the patient-level total score. The score was then treated as a diagnostic marker for post-LSG GERD and a non-parametric receiver operating characteristic (ROC) curve was generated. Two integer cut-offs that maximized the Youden index (sensitivity + specificity − 1) defined low-, intermediate-, and high-risk strata; the same routine in 2000 bootstrap resamples confirmed the stability of these thresholds.

Model performance was evaluated in development and validation cohorts by discrimination (DCA), calibration plots with Hosmer–Lemeshow test, and decision-curve analysis for clinical net benefit.

## Result

### General characteristics of the patients

A total of 851 patients met the inclusion criteria and were included in the final analysis, with 595 allocated to the training set and 256 to the validation set (Fig. 1). The mean age of the cohort was 31.77 ± 8.7 years, and the mean BMI was 38.39 ± 6.05 kg/m^2^. Females constituted 63.22% of the population. Preoperative reflux symptoms consistent with GERD were reported by 27.26% of patients, while GERDQ screening indicated low-grade GERD symptoms in 95.65% of the cohort. Other comorbid conditions included chronic constipation (6.58%) and ulcer disease (1.18%). No significant differences were found between the training and validation cohorts across the majority of demographic and clinical variables (all *P* > 0.05), indicating comparability between the two groups (Table 1).

**Table 1:** Demographic data and clinical characteristics of the training and validation sets

	Total N = 851	Training set N = 595	Validation setN = 256	*P* value
Age (year)	31.77 ± 8.7	31.57 ± 8.47	32.24 ± 9.2	0.30
Sex (female)	538 (63.22)	380 (63.87)	158 (61.72)	0.04
BMI (kg/m^2^)	38.39 ± 6.05	38.28 ± 6.14	38.65 ± 5.84	0.41
Waist (cm)	116.82 ± 15.08	116.64 ± 14	117.24 ± 17.37	0.59
Hip (cm)	120.21 ± 11.16	120.14 ± 11.03	120.37 ± 11.47	0.79
TWL (%)	20.06 ± 6.84	20.04 ± 6.77	20.11 ± 7.02	0.89
Smoking (n, %)	179 (21.03)	118 (19.83)	61 (23.83)	0.22
Drinking (n, %)	89 (10.46)	64 (10.76)	25 (9.77)	0.76
Surgery history (n, %)	187 (21.97)	130 (21.85)	57 (22.27)	0.96
NSAIDs (n, %)	28 (3.29)	19 (3.19)	9 (3.52)	0.97
Preoperative PPIs therapy (n, %)	12 (1.41)	11 (1.85)	1 (0.39)	0.18
Diabetes (n, %)	239 (28.08)	177 (29.75)	62 (24.22)	0.12
Hypertension (n, %)	375 (44.07)	252 (42.35)	123 (48.05)	0.14
Coach (n, %)	40 (4.70)	25 (4.20)	15 (5.86)	0.38
Chronic constipation (n, %)	56 (6.58)	44 (7.39)	12 (4.69)	0.19
HP (n, %)	131 (15.39)	89 (14.96)	42 (16.41)	0.66
Anemia (n, %)	48 (5.64)	35 (5.88)	13 (5.08)	0.76
Ulcer (n, %)	10 (1.18)	6 (1.01)	4 (1.56)	0.73
GERDQ	0 (0-12)	0 (0-12)	0 (0-12)	0.81
Distance from the pylorus (cm)
2–3	35 (4.11)	24 (4.03)	11 (4.30)	0.60
3–4	773 (90.83)	538 (90.42)	235 (91.80)
5–6	43 (5.05)	33 (5.55)	10 (3.91)
Distance from the angle of His (cm)		
>1	326 (38.31)	224 (37.65)	102 (39.84)	0.60
≤1	525 (61.69)	371 (62.35)	154 (60.16)
Duration of PPIs (weeks)	
≤12	410(48.18)	293(49.24)	117(45.70)	0.17
>12	441 (51.82)	302 (50.76)	139 (54.30)
Pre-op reflux-like symptoms (%)	150 (17.63)	104 (17.48)	46 (17.97)	0.94
AFS grade	
I	630 (74.03)	436 (73.28)	194 (75.78)	0.50
II	221 (25.97)	159 (26.72)	62 (24.22)
GERD (%)	232 (27.26)	160 (26.89)	72 (28.12)	0.77

BMI, body mass index; NSAIDs, non-steroidal anti-inflammatory drugs; TWL, total weight loss; PPIs, proton pump inhibitors; HP, helicobacter pylori; AFS, American Foregut Society; GERDQ, Gastroesophageal Reflux Disease Questionnaire.

Pre-op reflux-like symptoms = GERD-Q score <8 but ≥1 episode of heartburn/regurgitation per week.

Median follow-up duration ranged from 13.3 to 14.5 months, and mean %EWL varied from 79.9 ± 26.5% to 84.9 ± 24.6%. The incidence of new-onset GERD was relatively consistent across centers, ranging from 22.6% to 28.8%. Serious adverse events were rare, with an overall leak rate of 0.2%, and no cases of re-operation or mortality observed. New-onset GERD developed in 6 of 61 patients with ≥1 postoperative complication (9.8%) compared to 226 of 790 patients without complications (28.6%) (Supplemental Digital Content Table S1, available at: http://links.lww.com/JS9/E931). Similarly, GERD occurred in 38 of 118 patients who achieved <50% EWL (32.2%) versus 194 of 733 patients who achieved adequate weight loss (26.5%) (Supplemental Digital Content Table S2, available at: http://links.lww.com/JS9/E931).

### Clinical predictor selection

**Figure 2.**
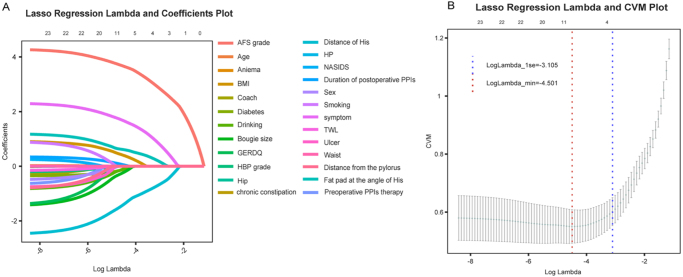
Selection of clinical features using the LASSO logistic regression model. (A) The curve representing partial likelihood deviance (binomial deviance) is shown against log(lambda). Vertical dashed lines indicate the optimal predictors based on the minimum criteria (min. criteria) and the 1 standard error of the minimum criteria (1-SE criteria). (B)LASSO coefficients for a total of 25 clinical features are displayed.

As shown in Table 2, Figure 2A, the LASSO model minimized partial likelihood deviance as a function of the regularization parameter (λ), allowing the progressive exclusion of non-contributory variables. Figure 2B illustrates the selection of the optimal λ value through 10-fold cross-validation, based on the minimum deviance and 1-standard-error (1-SE) criteria. Using this optimal threshold, 11 variables with non-zero coefficients were selected for inclusion in the final multivariable logistic regression model.

**Table 2:** Screening out risk factors for GERD by logistic regression

Variables		OR (95%CI)	*P* value
BMI (kg/m^2^)	>35 vs. ≤35	1.63(1.10–2.45)	0.017
Drinking	Yes vs. no	0.90(0.48–1.60)	0.718
Diabetes	Yes vs. no	0.66(0.43–1.00)	0.053
AFS grade	II vs. I	1.32(1.23–1.89)	0.021
Pre-op reflux-like symptoms	Yes vs. no	1.61(1.33–2.45)	0.005
Distance from the angle of His (cm)	>1 vs. ≤1	0.78(0.57–0.89)	0.042
Complete mobilization of the angle of His	Yes vs. no	1.25(1.14–1.79)	0.032
Bougie size (Fr)	<29	Ref	
	29–36	0.61(0.38–1.68)	0.442
	>36	0.67(0.37–1.63)	0.538
Duration of PPIs (weeks)	≤12	Ref	
	>12	0.57(0.38–0.98)	0.041

BMI, body mass index; OR, odds ratios; PPIs, proton pump inhibitors; AFS, American Foregut Society.

Pre-op reflux-like symptoms = GERD-Q score <8 but ≥1 episode of heartburn/regurgitation per week.

### Development and assessment of the predictive nomogram

**Figure 3.**
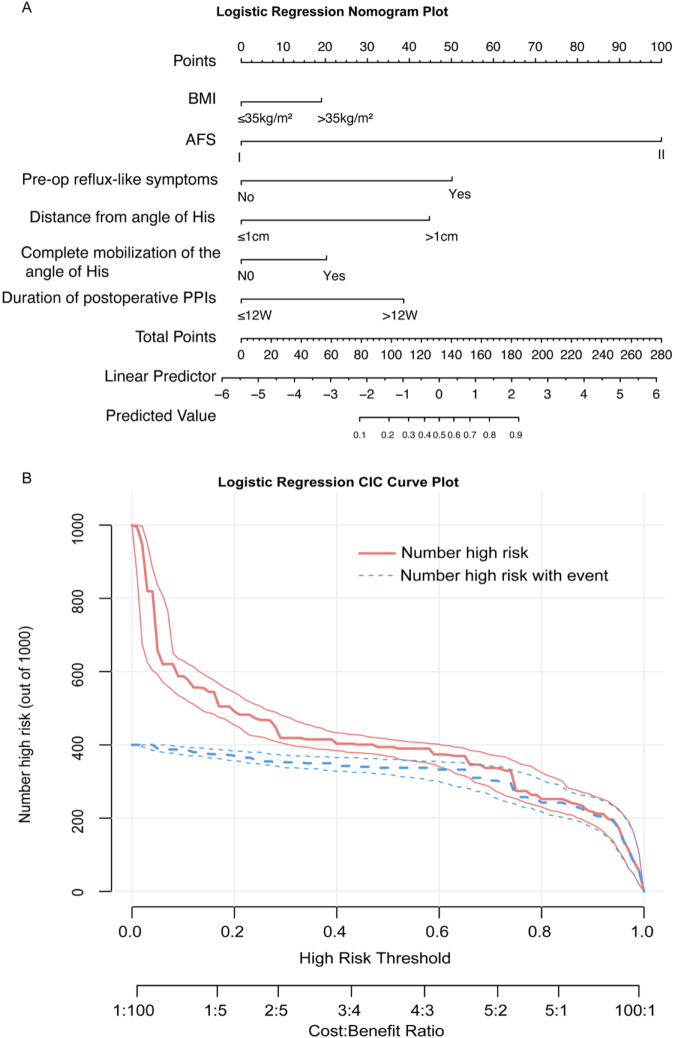
Nomogram and clinical impact curve for the predictive model. (A) Logistic regression-based nomogram for predicting the risk of postoperative GERD, BMI, AFS grade, preoperative reflux-like symptoms, staple-line distance from the angle of His, complete mobilization of the angle of His, and duration of postoperative PPI use. (B) Clinical impact curve (CIC) demonstrating the estimated number of patients classified as high risk and the number of true positive cases across different risk thresholds, illustrating the potential clinical utility of the model.

A nomogram was constructed based on the multivariable logistic regression model to estimate the probability of new-onset GERD after laparoscopic sleeve gastrectomy (LSG) (Fig. 3A). The model incorporated six significant predictors: BMI (>35 kg/m^2^), AFS grade II, presence of preoperative reflux symptoms, distance from the staple line to the angle of His (>1 cm), complete mobilization of the fat pad at the angle of His, and postoperative PPI duration. Each variable contributed a specific number of points, with AFS grade II contributing the highest (10 points), followed by reflux symptoms (5 points), BMI >35 kg/m^2^ and fat pad mobilization (2 points each), and shorter PPI duration (4–12 weeks) reducing the score (–2 points). The total point score was then used to estimate the predicted probability of GERD on a continuous scale.

### Clinical utility

To evaluate the model’s potential clinical benefit, a clinical impact curve (CIC) was plotted (Fig. 3B). The CIC showed that at a threshold probability of 0.4, approximately 410 out of 1000 patients would be classified as high risk, among whom ~320 were true positives. As the threshold increased, the number of patients identified as high risk decreased, while the model maintained reasonable discrimination between true and false positives. These findings suggest that the model may be useful for identifying patients at higher risk of GERD postoperatively and may help guide personalized follow-up strategies.

Each variable was assigned a weighted score according to its regression coefficient. For example, BMI >35 kg/m^2^ contributed 2 points, AFS grade II added 8 points, presence of preoperative reflux-like symptoms added 4 points, Complete resection of the fat pad added 1 point. Based on total scores, patients were stratified into three risk categories: low risk (≤1 points), medium risk (1 to 4 points), and high risk (>4 points) (Table 3).

**Table 3:** GERD risk grading system predicted probability of post-SG patients at 1 year

Variables	Point
BMI (kg/m^2^)	
≤35	0
>35	1
AFS grade	
I	0
II	8
Pre-op reflux-like symptoms	
No	0
Yes	4
Distance from the angle of His (cm)	
≤1	0
>1	−4
Complete mobilization of the angle of His	
No	0
Yes	1
Duration of PPIs (weeks)	
≤12	0
>12	−1

BMI, body mass index; TWL, total weight loss; PPIs, proton pump inhibitors; AFS, American Foregut Society.

Patients were divided into three groups according to the risk score (low risk, ≤1 points; medium risk, 1–4 points; high risk, >4 points).

### Model testing

In the training set, the model demonstrated excellent discrimination with an area under the ROC curve of 0.948, while the validation set yielded an AUC of 0.912 (Supplemental Digital Content Figure S1, available at: http://links.lww.com/JS9/E931), confirming strong predictive accuracy and model robustness. Calibration plots showed close agreement between the predicted and observed probabilities of GERD in both datasets, indicating good calibration (Supplemental Digital Content Figure S2, available at: http://links.lww.com/JS9/E931). Decision curve analysis further supported the clinical utility of the model by demonstrating a positive net benefit across a range of threshold probabilities (Supplemental Digital Content Figure S3, available at: http://links.lww.com/JS9/E931), suggesting the nomogram could aid clinical decision-making by identifying patients at elevated risk of postoperative GERD.

**Figure 4.**
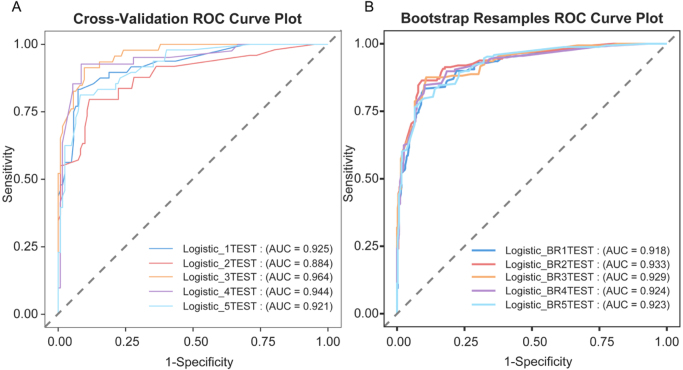
Internal validation of the predictive model using cross-validation and bootstrap resampling. (A) ROC curve based on 5-fold cross-validation showing the model’s discrimination performance. (B) ROC curve derived from 1000 bootstrap resamples, illustrating optimism-corrected discrimination ability.

Internal validation demonstrated good model performance across both methods. The mean cross-validated AUROC was 0.927, and the bootstrap-corrected AUROC was 0.926 (95% CI: 0.914–0.937) (Fig. 4), indicating minimal optimism. Other key metrics, including accuracy and calibration, also showed stable results, supporting the robustness of the model (Supplemental Digital Content Tables S3 and S4, available at: http://links.lww.com/JS9/E931).

To promote ease of use, we have deployed the model as an open-access web calculator (https://lsg-gerd.shinyapps.io/GERD), allowing clinicians to obtain patient-specific GERD risk estimates.

## Discussion

LSG has become the most widely performed bariatric surgery; however, controversy persists regarding whether it induces GERD^[23–25]^. This study conducted a detailed assessment of preoperative and postoperative risk factors for GERD in patients undergoing LSG, based on clinical symptoms, using questionnaires combined with endoscopic examinations before and after surgery. We found that within 1 year postoperatively, approximately 21% of patients reported new-onset GERD symptoms, a proportion similar to results reported in other studies^[26]^. Our multicenter retrospective study identified BMI, preoperative reflux-like symptoms, structural integrity of the EGJ observed under endoscopy, and the distance from the resection margin to the angle of His, used as significant influencing factors for the development of GERD after LSG.

A high preoperative BMI is an independent predictor for the occurrence of GERD after LSG. The higher the patient’s preoperative BMI, the greater the risk of postoperative GERD. Although patients with higher BMI experience weight loss after surgery, their BMI remains relatively elevated during follow-up. Due to increased intra-abdominal pressure, alterations in the structure of the EGJ, and weakened anti-reflux barrier function, gastric contents are more likely to reflux into the esophagus postoperatively, leading to GERD. This finding is consistent with previous studies^[27]^, suggesting that high BMI is an independent risk factor for GERD. Patients with preoperative reflux-like symptoms have a higher risk of developing GERD postoperatively. Some patients experience acid regurgitation and heartburn but do not meet the diagnostic criteria for GERD in questionnaire surveys. Preoperative reflux-like symptoms may reflect an existing pathological reflux state, indicating a higher risk of postoperative GERD. Current guidelines point out that severe esophagitis (LA-C or LA-D grade) and GERD with BE are contraindications for LSG^[28]^, but they do not cover cases where patients have only reflux-like symptoms. Therefore, identifying, evaluating, and managing preexisting preoperative reflux-like symptoms during preoperative screening may be important factors in reducing the risk of postoperative GERD, while regular postoperative monitoring is also required.

Studies have confirmed that disruption of the integrity of the EGJ is one of the important causes of GERD^[29]^. The EGJ is composed of the lower esophageal sphincter (LES) and the crural diaphragm. Our study also found that abnormalities in the structural integrity of the EGJ observed under endoscopy (EGJ grade II) significantly increase the risk of postoperative GERD. This may be due to the long-term increased intra-abdominal pressure in obese patients leading to anatomical abnormalities of the EGJ, reducing the function of the anti-reflux barrier and thus causing GERD symptoms. Additionally, during LSG surgery, the distance from the resection margin to the angle of His is an important factor affecting the incidence of postoperative GERD. The shorter the distance of the resection margin, the higher the incidence of postoperative GERD. Generally, maintaining a distance of 1–2 cm from the resection margin to the angle of His reduces the occurrence of postoperative GERD, possibly because a closer resection margin disrupts the integrity of the EGJ structure.

Whether calibration-bougie diameter is an independent predictor of de-novo GERD after sleeve gastrectomy remains unsettled^[30,31]^. A systematic review and meta-analysis demonstrated that the use of a smaller bougie was associated with a significantly higher percentage of excess weight loss (%EWL) compared to larger bougie sizes, while no statistically significant difference in the incidence of GERD was observed between the two groups^[32]^. Several recent analyses have linked smaller bougies (<36 Fr) to higher rates of postoperative GERD, presumably because tighter luminal restriction increases intragastric pressure; a network meta-analysis of 35 studies (13 450 patients) reported a 76% relative rise in GERD with 32–36 Fr bougies compared with ≥40 Fr bougies. In our study, the bougie size predominantly ranged from 34 to 38 Fr, representing a relatively narrow interval of variation. Moreover, variations in staple line reinforcement practices across different centers may have had a potential impact, contributing to the lack of a statistically significant difference. Consequently, it did not enter the final multivariable model. Currently, most scholars believe that choosing a bougie diameter between 32 and 36 French achieves a good balance between efficacy and complications. Given these conflicting data, larger prospective studies that purposely randomize or stratify bougie size are necessary before definitive practice recommendations can be made.

Our results highlight two important and modifiable surgical predictors of GERD following LSG: complete mobilization of the fat pad at the gastroesophageal junction and the staple-line distance from the angle of His. Adequate mobilization of the gastroesophageal fat pad disrupts the anatomical integrity of the anti-reflux mechanism. For example, Braghetto *et al* reported that complete mobilization compromises the natural flap-valve mechanism, increasing acid exposure and the risk of postoperative GERD^[33,34]^. Similarly, we observed that a staple line positioned too close to the angle of His (<1 cm) was associated with a higher risk of GERD, supporting previous studies that adequate preservation of gastroesophageal junction function maintain normal gastric anatomy and reduces postoperative reflux^[35]^. Taken together, these results underscore the importance of a standardized surgical technique – ensuring full fat-pad mobilization and appropriate staple-line placement – to minimize GERD after sleeve gastrectomy.

Our findings suggest that extending PPI therapy beyond 12 weeks postoperatively may independently reduce the risk of GERD. This is consistent with previous large registry studies reporting high rates of chronic PPI use among sleeve gastrectomy patients^[36]^. Physiologically, prolonged acid suppression may help protect against early mucosal injury and postoperative reflux^[37]^. However, the potential benefits of extended PPI use must be weighed against known risks, such as nutritional deficiencies and microbiome alterations. Further prospective studies are needed to define the optimal duration of PPI therapy, balancing clinical efficacy with long-term safety.

In summary, LSG is an effective bariatric surgery, but the potential risk of postoperative GERD warrants attention. Particularly for patients with a high preoperative BMI, preexisting typical GERD symptoms, and structural abnormalities of the EGJ, thorough and detailed preoperative assessment and obtaining informed consent are of significant value in choosing the surgical approach. Despite the strengths of our study, several limitations should be acknowledged. A notable limitation is the reliance on subjective GERD-Q assessment and routine endoscopy performed without standardized PPI discontinuation, which may lead to diagnostic inaccuracies. Future studies should incorporate objective evaluations such as 24-hour pH-impedance monitoring or esophageal manometry. Second, although the study included a large, multicenter cohort, the distribution of patients and surgical techniques across centers may have introduced inter-institutional variability. Finally, although the predictive model demonstrated high discrimination, external validation in prospective and diverse populations is necessary before clinical implementation. Future studies should incorporate prospective designs, long-term follow-up, and functional diagnostic tools to further elucidate the pathophysiology of post-LSG GERD and refine risk stratification for surgical decision-making.

## Conclusion

We developed and externally validated a risk scoring model for predicting the likelihood of GERD following LSG. The model incorporates relevant clinical and anatomical variables and showed good discriminative ability in both the training and validation cohorts. While these findings are promising, further prospective validation is needed before clinical application. The model may help inform preoperative evaluation and guide future research on GERD prevention after LSG.

## Data Availability

The data are available from the corresponding author on reasonable request.

## Research registration unique identifying number (UIN)

This study involving human participants was registered in a publicly accessible database in accordance with the World Medical Association’s Declaration of Helsinki (2013, Article 35). The registration was completed in the Research Registry, and the unique identifying number (UIN) is researchregistry11054.
